# Butyrate-mediated autophagy inhibition limits cytosolic *Salmonella* Infantis replication in the colon of pigs treated with a mixture of *Lactobacillus* and *Bacillus*

**DOI:** 10.1186/s13567-020-00823-8

**Published:** 2020-08-05

**Authors:** Bingxin Chu, Yaohong Zhu, Jinhui Su, Bing Xia, Yunjing Zou, Jiawei Nie, Wei Zhang, Jiufeng Wang

**Affiliations:** 1 College of Veterinary Medicine, China Agricultural University, No. 2 Yuanmingyuan West Road, Beijing, 100193 People’s Republic of China; 2 Institute of Animal Husbandry and Veterinary Medicine, Beijing Academy of Agriculture and Forestry Sciences, No. 9 Shuguanghuayuan Middle Road, Beijing, 100097 People’s Republic of China

**Keywords:** *Salmonella*, butyrate, autophagy, microbiota, probiotic, colon

## Abstract

Probiotics as an effective and safe strategy for controlling *Salmonella* infection are much sought after, while autophagy is a central issue in eliminating intracellular pathogens of intestinal epithelial cells. In this study, an animal model of colitis has been developed by infecting weaned pigs orally with a strain of *Salmonella* Infantis in order to illuminate the potential efficacy of a mixture of *Lactobacillus* and *Bacillus* (CBB-MIX) in the resistance to *Salmonella* infection by regulating butyrate-mediated autophagy. We found that CBB-MIX alleviated *S*. Infantis-induced colitis and tissue damage. Autophagy markers ATG5, Beclin-1, and the LC3-II/I ratio were significantly enhanced by *S*. Infantis infection, while treatment with CBB-MIX suppressed *S*. Infantis-induced autophagy. Additionally, *S*. Infantis-induced colonic microbial dysbiosis was restored by this treatment, which also preserved the abundance of the butyrate-producing bacteria and the butyrate concentration in the colon. A Caco-2 cell model of *S*. Infantis infection showed that butyrate had the same effect as the CBB-MIX in restraining *S*. Infantis-induced autophagy activation. Further, the intracellular *S*. Infantis load assay indicated that butyrate restricted the replication of cytosolic *S*. Infantis rather than that in *Salmonella*-containing vacuoles. Suppression of autophagy by knockdown of ATG5 also attenuated *S*. Infantis-induced cell injury. Moreover, hyper-replication of cytosolic *S*. Infantis in Caco-2 cells was significantly decreased when autophagy was inhibited. Our data demonstrated that *Salmonella* may benefit from autophagy for cytosolic replication and butyrate-mediated autophagy inhibition reduced the intracellular *Salmonella* load in pigs treated with a probiotic mixture of *Lactobacillus* and *Bacillus.*

## Introduction

*Salmonella enterica* serovar Infantis (*S*. Infantis), one of the most prevalent *Salmonella* serovars [1, 2], is of particular concern as it is a zoonotic pathogen usually transferred via contaminated food products [3–5]. *S*. Infantis is a non-typhoidal serovar, which usually causes self-limiting inflammation of the host gut [4, 6]. As a facultative intracellular pathogen, after invasion of epithelial cells, *S*. Infantis resides within a membrane-bound compartment referred to as the *Salmonella*-containing vacuole (SCV) [7, 8]. Although the SCV is considered to be the intracellular niche for *Salmonella*, its low pH and nutrient-limited environment are not suitable for bacterial survival [8–10]. Recent findings indicate that *Salmonella* within SCV can escape into the cytoplasm where replication far exceeds that within SCV. This phenomenon is known as hyper-replication (defined as 50 bacteria per cell), a state where *Salmonella* expresses SPI-1 genes and synthesize flagella in preparation for further invasion [8, 11, 12]. These bacteria then pass through infected cells to escape into the interstitium, where they can cause infections in neighboring cells that eventually spread through the enteric cavity [12].

Autophagy is a highly conserved biological process in eukaryotes, which targets intracellular components to lysosomes for degradation [13, 14]. Autophagy also serves as a natural immune mechanism, known as xenophagy, to eliminate bacteria that invade cells [15–17]. Paradoxically, autophagy seems to play the opposite role in the process of *Salmonella* infection. One earlier study showed that suppression of GTPase expression, which is necessary for autophagy, reduced *Salmonella* replication in HeLa cells [18]. Additional research had similar findings that knockdown of LC3 and p62 limited *Salmonella* proliferation [19]. Some pathogens have developed countermeasures against the cellular defense response, such as *Shigella flexneri*, which can restrain autophagosome generation by secreting the effector protein IcsB [20]. Furthermore, our previous study showed that *Lactobacillus rhamnosus* GG controlled *S*. Infantis infection by suppressing activation of intestinal epithelial cell autophagy in pigs [21]. More recently, we found that *L. johnsonii* prevented *S*. Infantis dissemination and ameliorated enteritis via suppression of autophagy in weaned pigs [22].

Gut microbiota is a critical factor in host resistance to pathogen infection. *Salmonella* causes intestinal inflammation and remodeling of gut microbiota, which leads to impaired colonization resistance and increases the susceptibility of the host to *Salmonella* [23]. Probiotics can protect the host from enteric infection by reprogramming the intestinal microbiota community and the efficacy of compound probiotics is superior to a single probiotic preparation [24, 25]. The colon has one of the most abundant microbiota of the entire intestinal tract, therefore, maintaining the stability of the host colonic microbiota is crucial for host wellbeing. Recent studies have confirmed that colonic microbiota maintains host health by producing short chain fatty acids (SCFAs) [26, 27]. While SCFAs are absorbed by colonic mucosa, butyrate is transported preferentially and seems to be the preferred energy source for colonic cells [28]. It has been proposed that an insufficient energy supply, 70% of which normally originates from butyrate, could be a causative factor for colitis [29]. Indeed, our previous study showed that oral administration of *L. johnsonii* maintained short-chain fatty acid levels and reduced the colonization of *S*. Infantis in the intestine [30]. Remarkably, butyrate can suppress autophagy at physiological concentration [29]. However, the underlying mechanism by which gut microbiota-targeted probiotics and butyrate control *S*. Infantis infection remains to be explored.

In the present study, we showed that a probiotic mixture of *Lactobacillus* and *Bacillus* maintained butyrate concentration by regulating gut microbiota during *S*. Infantis infection. Furthermore, butyrate-mediated autophagy inhibition reduced the intracellular *Salmonella* load and resisted *Salmonella* infection in pigs treated with a probiotic mixture of *Lactobacillus* and *Bacillus*.

## Materials and methods

### Bacterial strains

*Salmonella enterica* serovar Infantis strain CAU1508 was isolated from the intestinal contents of weaned piglets with diarrhea in our laboratory. *S*. Infantis harboring the pFPV-mCherry or pFPV25.1 plasmid was prepared as previously described [31]. *S*. Infantis carrying the pFPV-mCherry plasmid or pFPV25.1 plasmid can express red or green fluorescent protein, respectively. *S*. Infantis was grown in LB medium overnight with shaking at 200 × *g* and 37 °C, followed by being subcultured (1:40) into 10 ml fresh LB medium for growth under the same conditions for 3.5–4 h. The bacteria were then centrifuged at 4000 × *g* for 15 min and resuspended in saline for in vivo experiments or PBS for in vitro experiments.

*Lactobacillus Johnsonii* (CJ21), *B. subtilis* (BS15), *B. licheniformis* (BL21) were isolated from the intestinal contents of healthy pigs in our laboratory. *L. johnsonii* CJ21 was grown in De Man, Rogosa, and Sharpe (MRS) broth (Oxoid, Basingstoke, UK) for 24 h at 37 °C under microaerophilic conditions. *B. subtilis* BS15 and *B. licheniformis* BL21 were grown in LB medium for 24 h at 37 °C under microaerophilic conditions. After overnight incubation, the bacteria were inoculated 1:100 into fresh MRS or LB broth and grown for 8 h until mid-log growth phase was reached.

### Animals and experimental design

A total of 28 healthy pigs (Landrace × Large White) weaned at 21 days of age were all obtained from the Beijing Hog Raising and Breeding Center (Beijing, China). Piglets were selected from seven different litters, with four piglets from each litter. These four piglets were divided into four different groups. All feces were collected for *Salmonella* antigen detection to ensure that the piglets were not infected with *Salmonella* prior to the experiment. Subsequently, the piglets were fed with antibiotic-free feed and water ad libitum for 3 days to adapt to the new environment. Then piglets were used in the experiment at 25 days of age. On day 0, pigs were randomly divided into four groups (n = 7 per group): (1) control (CONT) group: intragastric administration of 10 mL/day sterile physiological saline for the first 7 days; (2) CBB-MIX group: intragastric administration of 10 mL/day CBB-MIX: *L. johnsonii* CJ21 1 × 10^9^ CFU/mL), *B. subtilis* BS15 (1 × 10^6^ CFU/mL), *B. licheniformis* BL21 (1 × 10^6^ CFU/mL) for the first 7 days; (3) SI group: intragastric administration of 10 mL/day physiological saline for the first 7 days and challenged with *S.* Infantis (5 × 10^10^ CFU/mL, 10 mL/day) on day 8; (4) CBB-MIX + SI group: intragastric administration of 10 mL/day CBB-MIX solution for the first 7 days and challenged with *S*. Infantis (5 × 10^10^ CFU/mL, 10 mL) on day 8. On day 10, one piglet from each group was slaughtered to determine whether the experimental model for colon damage caused by *Salmonella* Infantis was successfully established. Finally, on day 13, all the pigs were euthanized and tissue samples were immediately collected.

### Reagents and antibodies

Lyso-Tracker Red (L8010), 4′,6′-diamidino-2-phenylindole (DAPI) solution (C0060), and Calcein-AM/PI (CA1630) were purchased from Beijing Solarbio Science & Technology Co., Ltd. (Beijing China). Chlorquine diphosphate salt (C6628), butyrate sodium (B5887), and Triton X-100 (T8787) were obtained from Sigma-Aldrich (St. Louis, USA). Dulbecco’s Modified Eagle Medium (DMEM)/High Glucose (SH30022.01) and phosphate buffered saline (PBS) were purchased from GE Healthcare Life Sciences HyClone Laboratories (Utah, USA). Fetal bovine serum (FBS) was obtained from Thermo Fisher Scientific (Rockford, USA). Lipofectamine™ RNAiMAX transfection reagent (13778075) was obtained from ThermoFisher Scientific (Rockford, USA). Conventional chemosynthetic siRNA for ATG5 was obtained from Living Biotechnologies Co., Ltd. The following primary antibodies: rabbit anti-ATG5 polyclonal antibody (10181-2-AP); rabbit anti-Beclin 1 polyclonal antibody (11306-1-AP); rabbit anti-p62/SQSTM1 polyclonal antibody (18420-1-AP); rabbit anti-LAMP 1 polyclonal antibody (21997-1-AP); mouse anti-LAMP 2 polyclonal antibody (66301-1-lg); rabbit anti-ATP6V1A polyclonal antibody (17115-1-AP); rabbit anti-ATP6V1B2 polyclonal antibody (15097-1-AP); rabbit anti-ATP6V1E1 polyclonal antibody (15280-1-AP); rabbit anti-Occludin polyclonal antibody (27260-1-AP); mouse anti-GAPDH monoclonal antibody (60004-1-lg); and mouse anti-Beta ACTIN monoclonal antibody (60008-1-lg) were purchased from Proteintech Group Inc (Rosemont, IL 60018, USA). Rabbit anti-LC3A/B polyclonal antibody (#4108) was obtained from Cell Signaling Technology (Danvers, MA 01923, USA). Rabbit anti-ZO-1 polyclonal antibody was purchased from ThermoFisher Scientific (Rockford, USA). Rabbit anti-Claudin-1 polyclonal antibody (ab15098) was obtained from abcam (Cambridge, UK). The secondary antibodies used for Western Blotting were purchased from Proteintech Group Inc: HRP-conjugated Affinipure Goat Anti-Rabbit IgG(H+L) (SA00001-2) and HRP-conjugated Affinipure Goat Anti-Mouse IgG(H+L) (SA00001-1). Alexa Fluor 488-labeled Goat Anti-Rabbit lgG(H+L) and Alexa Fluor 555-labeled Donkey Anti-Rabbit lgG(H+L) were obtained from Beyotime Biotechnology (Shanghai, China).

### Histopathologic scoring

To assess intestinal pathology, mid-segments (10 × 15 × 3 mm) of the colon were excised, rinsed with saline and then immersed in 4% paraformaldehyde. Paraffin-embedded samples of the colon tissues were sectioned (3 μm) and stained with hematoxylin and eosin. The level intestinal inflammation was scored as previously described [32].

### Western blotting

Total proteins were extracted from the middle segment of the colon or each well of Caco-2 cells using RIPA buffer (Solarbio, Beijing, China) containing a protease/phosphatase inhibitor cocktail (Cell Signaling Technology, USA). Protein concentration was quantified using a BCA Protein Assay kit (23227, ThermoFisher Scientific), prior to separation by sodium dodecyl sulfate-polyacrylamide gel electrophoresis and transfer onto polyvinylidene fluoride membranes. After blocking the membranes with 5% skim milk at room temperature for 1.5 h and incubation at 4 °C overnight, different membranes were incubated with the following corresponding primary antibodies: anti-ATG5 (1:500), anti-Beclin 1 (1:1000), anti-p62/SQSTM1 (1:2000), anti-LAMP 1 (1:2000), anti-LAMP 2 (1:1000), anti-ATP6V1A (1:1000), anti-ATP6V1B2 (1:2000), anti-ATP6V1E1 (1:2000), anti-occludin (1:1500), anti-GAPDH (1:5000), anti-beta ACTIN (1:5000), anti-LC3A/B (1:1000), anti-ZO-1 (1:250) and anti-claudin-1 (1:2000). Membranes were then subjected to horseradish peroxidase-conjugated secondary antibodies (1:5000) raised against the corresponding species source for the primary antibody for 1 h at room temperature. Then ECL western blotting substrate (Tanon Science & Technology Co., Ltd. Shanghai, China) was placed on the membranes. The images were obtained with a Tanon 6200 chemiluminescence imaging workstation (Tanon Science & Technology Co., Ltd. Shanghai, China). Each test was performed in triplicate.

### Immunofluorescence

Colonic samples were fixed in 4% formaldehyde for 24 h and embedded in paraffin prior to sectioning. The sections were dewaxed, rehydrated, and subjected to microwave antigen retrieval. Caco-2 cells were treated as described above. Samples were fixed with 4% paraformaldehyde for 7 min and permeabilized with PBS for 13 min. In the following step, all the tissue and cell samples were blocked with 2% bovine serum albumin for 1.5 h at room temperature. Then samples were incubated with anti-LAMP1 antibody (1:100), anti-ZO-1 antibody (1:50), anti-claudin-1 antibody (1:1000), and anti-occludin antibody (1:200) at 4 °C overnight. After washing three times with PBS, samples were incubated with Alexa Fluor 488 goat anti-rabbit or Alexa Fluor 555 donkey anti-rabbit secondary antibodies at room temperature for 1 h. DAPI was used to stain the nuclei for 10 min at room temperature. Images were obtained on a Nikon A1 confocal laser scanning microscope. At least 100 infected cells were counted for each independent experiment.

### Microbial genomic DNA extraction and 16S rRNA gene amplification

Samples of colonic contents (from middle segment of the colon) were collected immediately after sacrifice. Total microbial genomic DNA was extracted using a QIAamp DNA Microbiome kit (Qiagen, Hilden, Germany) according to the manufacturer’s instructions and the purified products checked on a 1% agarose gel, prior to the DNA concentration and purity being determined with a NanoDrop 2000 UV–vis spectrophotometer (Thermo Scientific, Wilmington, USA).

The hypervariable region V3–V4 of the bacterial 16S rRNA gene was amplified with primer pairs 338F (5′-ACTCCTACGGGAGGCAGCAG-3′) and 806R (5′-GGACTACHVGGGTWTCTAAT-3′) in an ABI GeneAmp 9700 PCR thermocycler (Applied Biosystems, CA, USA). The PCR amplification procedure was performed as follows: initial denaturation at 95 °C for 3 min, followed by 30 cycles of denaturing at 95 °C for 30 s, annealing at 55 °C for 30 s and extension at 72 °C for 45 s, and single extension at 72 °C for 10 min, and subsequently kept at 10 °C. The PCR mixtures contained 4 μL 5 × TransStart FastPfu buffer, 2 μL dNTPs (2.5 mM), 0.8 μL forward primer (5 μM), 0.8 μL reverse primer (5 μM), 0.4 μL TransStart FastPfu DNA Polymerase, 10 ng of template DNA, and lastly ddH_2_O to a final volume of 20 μL. PCR reactions were performed in triplicate and then the three PCR products were mixed for the electrophoresis on a 2% agarose gel. The specific 468-bp PCR products were extracted and purified using an AxyPrep DNA Gel Extraction kit (Axygen Biosciences, Union City, USA), and quantified using a Quantus™ Fluorometer (Promega, Madison, USA) according to the manufacturer’s instructions, prior to sequencing as described below.

## 16S rRNA gene sequencing and bioinformatics analysis

Purified 16S rRNA gene amplicons were pooled in equimolar concentrations and paired-end sequenced (2 × 300 bp) on an Illumina MiSeq platform (Illumina, San Diego, USA) according to the standard protocols provided by Majorbio Bio-Pharm Technology Co. Ltd. (Shanghai, China). The raw 16S rRNA gene sequencing reads were demultiplexed, quality-filtered using Trimmomatic and merged by FLASH with the following criteria: (i) the 300 bp reads were truncated at any site receiving an average quality score of < 20 over a 50 bp sliding window, and truncated reads shorter than 50 bp and reads containing ambiguous characters were also discarded; (ii) only overlapping sequences longer than 10 bp were assembled according to their overlapped sequence, where the maximum mismatch ratio of the overlap region was 0.2 and reads that could not be assembled were discarded; and (iii) samples were distinguished according to the barcode and primer sequences, and the sequence direction was adjusted. Note that exact barcode matching and a maximum of two nucleotide mismatches in the primer sequences were required for use. Operational taxonomic units (OTUs) with 97% similarity cutoff were clustered using UPARSE (version 7.1, http://drive5.com/uparse/), and chimeric sequences were identified and removed. The taxonomy of each OTU representative sequence was analyzed by the Ribosomal Database Project (RDP) Classifier (http://rdp.cme.msu.edu/) against the Silva SSU128 database using a confidence threshold of 70%. The alpha diversity (Shannon and Simpson), richness (ACE and Chao1), rarefaction curve, and Venn diagram analysis were performed using Mothur version 1.31.2 (http://www.mothur.org). A heatmap was created based on the relative abundance of the OTUs using R software (version 2.15) (http://www.R-project.org). Multivariate data analysis, including partial least squares-discriminate analysis (PLS-DA) and principle coordinate analysis (PCoA) based on unweighted unifrac distance, was performed using Simca-P 12.0 (Umetrics, Umea, Sweden) and R (version 3.2.1), respectively.

### Quantification of SCFAs

The concentrations of acetate, propionate, butyrate, isobutyrate, isovalerate, and valerate in the colonic contents were determined using gas chromatography (Agilent 7890 A, Agilent Technologies, Santa Clara, USA) as previously described [33].

### Cell culture and infection

1 × 10^6^ cells (per well) were seeded in 6-well culture plates, and 1 × 10^5^ cells (per well) were seeded in 24-well culture plates. Caco-2 cells were cultured in DMEM/High Glucose media supplemented with 10% FBS and 1% penicillin streptomycin at 37 °C in a 5% CO_2_ incubator. The butyrate was diluted to 10 mM in DMEM medium. The cells were incubated with 10 mM butyrate for 2 h and then washed three times with PBS to remove the residual butyrate. *S*. Infantis was diluted in medium and added at a multiplicity of infection (MOI) of 50. After 30 min, the cells were washed three times with PBS to remove non-internalized bacteria. Subsequently, the cells were cultured in fresh growth medium containing 100 mg/mL gentamicin for 2 h to kill any remaining extracellular *S*. Infantis, followed by replacing this with growth medium containing 10 mg/mL gentamicin to restrict the extracellular growth of *S*. Infantis for the remaining incubation time until sample collection. Butyrate was not added to the medium during the infection, however, after the medium was replaced post-infection, all reagents contained 10 mM butyrate. Three replicates were conducted for each cell sample.

### Scanning electron microscopy (SEM)

Caco-2 cells were collected at the designed time points and fixed with 3% glutaraldehyde for 48 h. The samples were treated using a standard SEM procedure, as previously described [34].

### Chloroquine (CQ) resistance assay

A CQ resistance assay was performed to obtain the quantity of cytosolic bacteria as a fraction of the total bacteria internalized into cells, as previously described [8, 11]. Cells were co-incubated with 700 μM CQ and 100 μg/mL gentamicin simultaneously for 1 h to quantify the number of cytosolic *S*. Infantis, while control cells were incubated with gentamicin only. At 0, 2, 4, 6, 8, 10, and 12 hpi, cells were washed three times with PBS and lysed in 200 μL PBS containing 0.3% Triton X-100. Bacteria released from cells were serial diluted and plated on LB agar plates (with 100 μg/mL ampicillin to select *S*. Infantis with pFPV-mCherry plasmid) to quantify the bacteria. Three independent replicates were conducted for each timepoint.

### Calcein-AM/PI cell mortality assay

Cells were seeded into 24-well plates in complete medium. At 8 hpi, all media was removed and 2 ml of 1× AssayBuffer was added to each well. The cell mortality was evaluated by double staining: 2 μM calcein-AM and 4.5 μM PI for 15 min at 37 °C. The viable and dead cells (yellow green fluorescence and red fluorescence respectively) were detected simultaneously with a 490 ± 10 nm excitation filter under a fluorescence microscope. Five visual fields were randomly selected and 100 cells were counted from each view. Finally, the percentage of cells with each color was calculated.

### RNA interference

Caco-2 cells were seeded in 24 well plates and grown to 60–80% confluence at the time of transfection. Cells were transfected with Atg5-siRNA diluted with the RNAiMAX transfection reagent according to the manufacturer’s instructions. At 5 h after transfection, the media was removed and cells were incubated with DMEM at 37 °C for 48 h.

### Data deposition and data bank accession number

The raw 16S rRNA gene sequences have been deposited into the US National Center for Biotechnology Information Sequence Read Archive database (accession number: PRJNA597824).

### Statistical analysis

Experiments were conducted at least three times. The statistical analysis was performed using GraphPad Prism 7 with a one-way ANOVA or a t-test with Bonferroni correction. Data were presented as mean ± SEM and a *P*-value < 0.05 was regarded as statistically significant. For data of relative abundance of taxa at the phylum, class, family, genus, and species levels from 16S rRNA gene sequencing, differences between the least-square means were compared using Tukey’s post hoc test with multiple testing corrections applied according to the Benjamini–Hochberg’s false discovery rate (*q* value), based on the global P values of the variables compared. A *P*-value of < 0.05 with a *q* value of < 0.05 was considered indicative of significance.

## Results

### Oral CBB-MIX alleviated the *S*. Infantis-induced colon injury in weaned pigs

**Figure 1:**
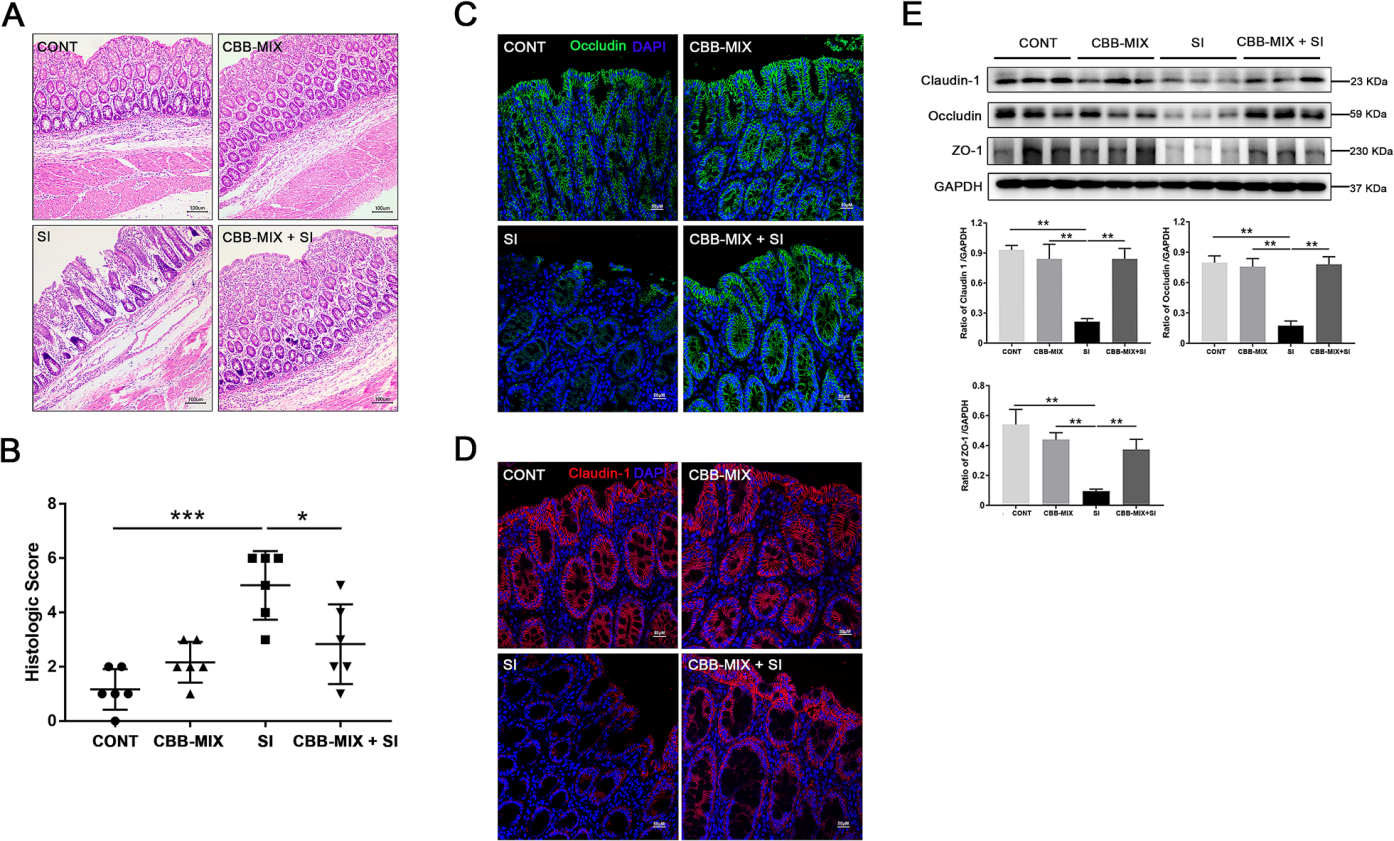
**Orally fed CBB-MIX alleviated the colon injury of weaned pigs caused by*S*. Infantis. A**, **B** Colon histological scores and representative photomicrographs of H&E stained section. **C**, **D** Detection of the distribution and expression of occludin (Green) and claudin-1 (Red) in colon tissue by immunofluorescence microscopy. Blue: DAPI. Scale bar, 50 µm. E, Western blot analysis for the expression levels of claudin-1, occludin and ZO-1 proteins in colon tissues from piglets. The lower panel shows the quantitation analysis by Image J software. All these data are presented as mean ± SEM (n = 6), *P < 0.05, **P < 0.01, ***P < 0.001.

To investigate whether the CBB-MIX plays a protective role against *S*. Infantis infection, we conducted a comprehensive histological analysis of the colon. First, histologic analysis showed that, compared with the CONT group, pigs in the SI group exhibited obvious pathological injury in the colon in the form of epithelial stripping and fissures; mucosa hyperemia; inflammatory cell infiltration; submucosal connective tissue loosening and hyperemia (Figure 1A). However, oral administration of CBB-MIX relieved the severity of *S*. Infantis-induced histopathological injury in the colon. While increased intestinal inflammation compared with the colon of CONT pigs (*P *< 0.001, Figure 1B) was observed in the SI group, lower histologic scores were observed in the colon of CBB-MIX + SI pigs compared with that of SI pigs (*P *= 0.01). The intestinal mucosal mechanical barrier is an important physiological barrier against invasion of pathogenic microorganisms [35, 36]. Immunofluorescence results indicated that *S*. Infantis challenge disrupted the tight junction structure of the epithelium and resulted in the fracture and disappearance of intercellular occludin (Figure 1C) and claudin-1 (Figure 1D) proteins. Oral administration of the CBB-MIX preserved the complete tissue structure and maintained the uniform distribution of intercellular occludin and claudin-1 during *S*. Infantis infection. Western blot analysis also demonstrated that compared with the CONT group, lower levels of claudin-1, occludin and ZO-1 proteins were observed in the SI group (*P *= 0.002, *P *= 0.001 and *P *= 0.002, respectively, Figure 1E). Conversely, higher levels of claudin-1, occludin and ZO-1 proteins were found in the CBB-MIX + SI group compared with the SI group (*P *= 0.002, *P *= 0.001 and *P *= 0.003, respectively, Figure 1E). These results indicated that oral administration of the probiotic CBB-MIX attenuated *S*. Infantis-induced colon injury in pigs.

### Orally fed CBB-MIX affected the colonic microbiota composition and maintained the abundance of butyrate-producing bacteria during *S*. Infantis infection

**Figure 2:**
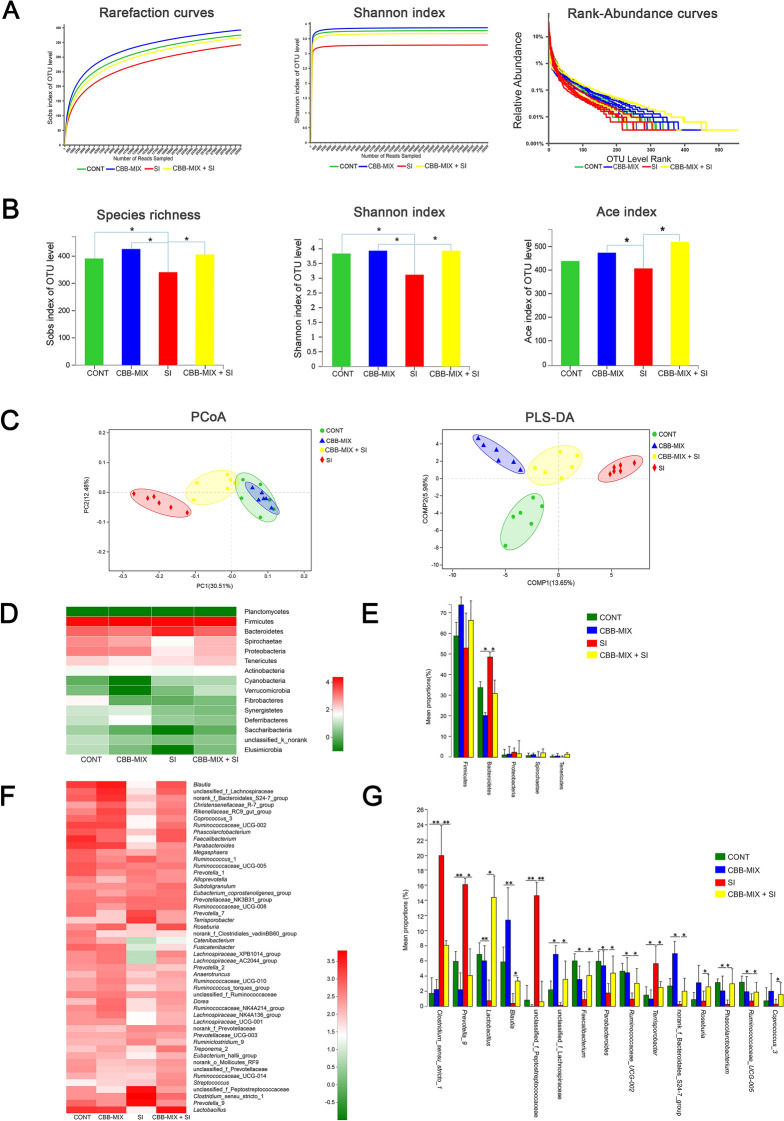
**Diversity analysis of colonic microbiota. A Diversity analysis of colonic microbiota.** The rarefaction abundance curves, Shannon index, and rank abundance curves were used to assess the colonic microflora diversity at 97% similarity in 4 groups (n = 6). **B** Difference analysis of alpha diversity index (Sobs, Shannon, and Ace index) between groups. Alpha diversity was illustrated by number of observed OTU using a one-way ANOVA. *P < 0.05. **C** Comparative analysis of colonic microbiota. Beta diversity was illustrated by Principal coordinate analysis (PCoA) of the overall microbial composition (Distance Algorithm: unweighted unifrac, ANOSIM R: 0.591, *P* = 0.001). Partial Least Squares Discriminant Analysis (PLS-DA) was used to represent sample grouping analysis results (Distance Algorithm: unweighted unifrac). **D** Heatmap analysis showed the relative abundance of the bacterial at the phylum level in four groups. **E** Differences in the relative abundance of genera from the 4 groups are shown using one-way ANOVA. *P < 0.05, **P < 0.01, ***P < 0.001. **F** Heatmap analysis showed the relative abundance of the top 50 genera in four groups. **G** Differences in relative abundance of genera from the 4 groups are shown using one-way ANOVA. All these data were presented as mean ± SEM. *P < 0.05, **P < 0.01.

High throughput 16S rRNA gene sequencing was performed to explore the effect of CBB-MIX on the colonic microbial community during *S*. Infantis infection. After optimizing the raw sequences, a total of 1,224,788 sequences from 24 samples were obtained for further analysis. The slopes of the rarefaction curves based on the Sobs and Shannon indices tended to be gentle (Figure 2A), indicating that the sequencing data had reached saturation and the sequencing depth reflected the vast majority of microbial diversity information. As shown in the rank-abundance curves (Figure 2A), a small number of OTUs dominated and the majority of OTUs were present at low abundance in the colonic microbiota. The rarefaction curves based on the Sobs, Shannon and rank abundance curves showed that the CONT, CBB-MIX, and CBB-MIX + SI groups had greater taxon richness than the SI group. One-way ANOVA tests showed that compared with the CONT group, a decreased number of OUT were observed (as shown by the Sobs and Shannon indices) in the SI group, but not in pigs from the CBB-MIX and CBB-MIX + SI groups (Figure 2B). Compared with the SI group, pigs in the CBB-MIX and CBB-MIX + SI groups had a higher number of OTU. The PCoA based on unweighted unifrac distance showed that the coordinate points in the CBB-MIX group were arranged closer and shorter than those in the other three groups, indicating the CBB-MIX could regulate the consistency of the colonic microbiota structure. The colonic microbiota of SI pigs was clearly distinguishable from the other three groups, and oral CBB-MIX shifted the overall structure of the *S*. Infantis-disrupted colonic microbiota toward that of the CONT and CBB-MIX pigs (Figure 2C). Additionally, the PLS-DA based on the unweighted unifrac distance also displayed similar results (Figure 2C), demonstrating that treatment with the CBB-MIX maintained the stability and diversity of colonic microbiota under disturbance by *S*. Infantis.

In total, 15 phyla were identified across all the samples (Figure 2D). The results of heat map indicated that the dominant phyla were the same in the different samples at the phylum level, with Firmicutes and Bacteroidetes accounting for the majority of the total sequences (Figure 2D). Compared with the CONT group, *S*. Infantis infection increased the abundance of Bacteroidetes (*P *= 0.034), but this increase was inhibited by treatment with the CBB-MIX (*P* = 0.046) (Figure 2E). Heat map analysis revealed the top 50 genera with relative abundance at the genus level (Figure 2F). Compared with the CONT group, there was an obvious shift in the colonic microbiota in the SI group, while the microbiota profiles in CBB-MIX and CBB-MIX + SI groups were similar to that in the CONT group (Figure 2F). The ANOVA results showed that compared with the CONT group, the genera *Prevotella*_9 (*P *= 0.002), *Clostridium_sensu_stricto*_1 (*P *= 0.004), unclassified_f_*Peptostreptococcaceae* (*P *= 0.010), and *Terrisporobacter* (*P *= 0.039) were markedly enriched in the SI group, whereas oral administration of CBB-MIX prevented this increase. Indeed, this treatment reversed the *S*. Infantis-induced decrease in the genera *Lactobacillus* (*P *= 0.039), *Blautia* (*P *= 0.016), unclassified_f_*Lachnospiraceae* (*P *= 0.049), *Faecalibacterium* (*P *= 0.027), *Parabacteroides* (*P *= 0.017), *Ruminococcaceae*_UCG-002 (*P *= 0.024), norank_f_*Bacteroidales*_S24-7_group (*P *= 0.013), *Roseburia* (*P *= 0.041), *Phascolarctobacterium* (*P *= 0.035), *Coprococcus*_3 (*P *= 0.026, Figure 2G). Of particular note was that the oral CBB-MIX treatment maintained the abundance of butyrate-producing bacteria, including *Lactobacillus*, *Blautia*, *Ruminococcaceae*_UCG-002, *Faecalibacterium*, and populations belonging to *Lachnospiraceae*.

### Effect of oral CBB-MIX on the level of SCFAs during *S*. Infantis infection

**Figure 3:**
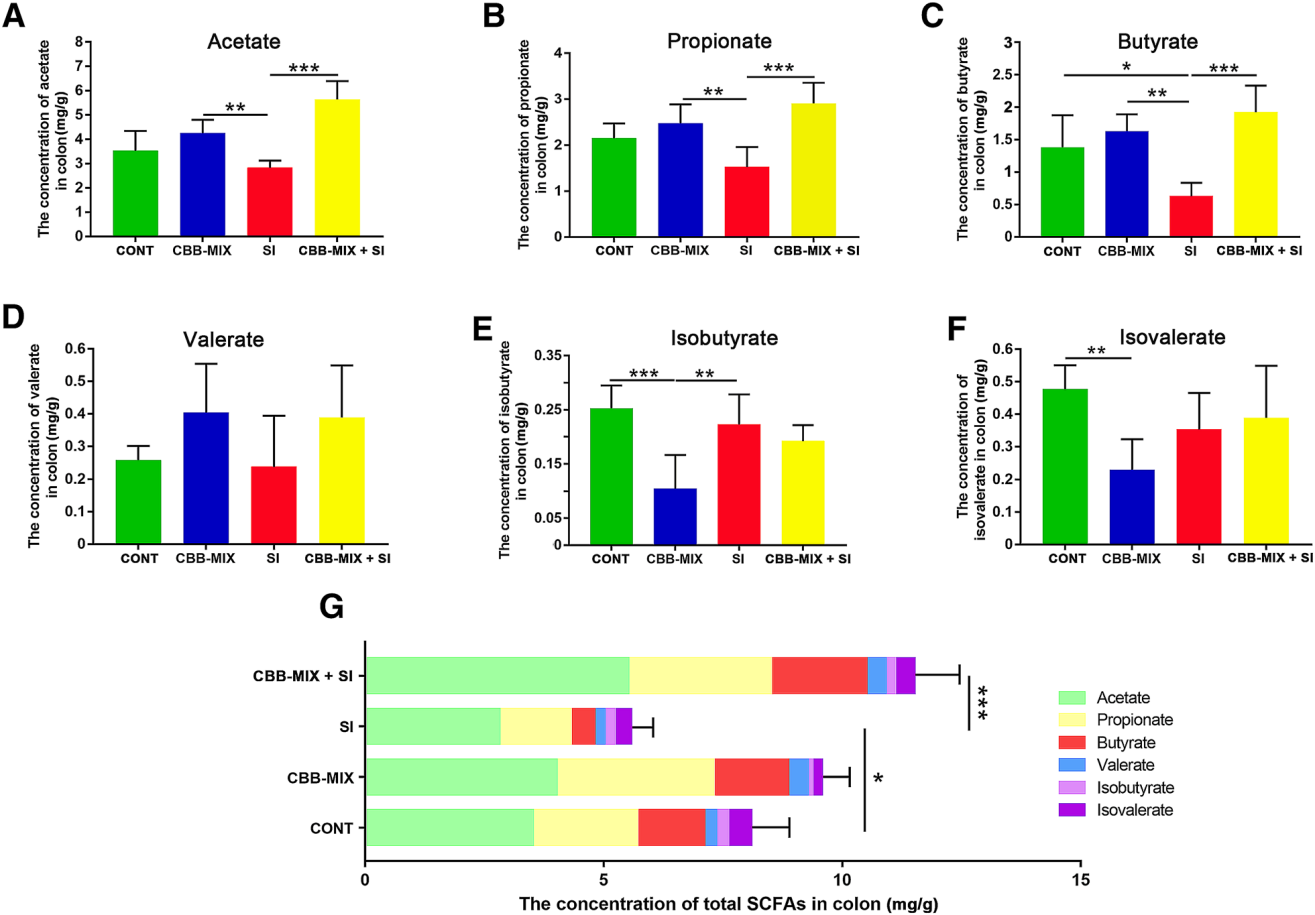
**The effect of oral administration of CBB-MIX on the level of SCFAs during*S*. Infantis infection. A**–**G** Concentrations of acetate, propionate, butyrate, valerate, isobutyrate, isovalerate, and total SCFAs levels in the colonic contents of pigs. The data were presented as mean ± SEM (n = 6), *P < 0.05, **P < 0.01. ***P < 0.001.

Next, the levels of each of five SCFAs were detected in the colon contents of pigs in response to *S*. Infantis infection alone or after CBB-MIX pretreatment. Acetate and propionate were found to be elevated in the CBB-MIX and CBB-MIX + SI groups, compared with the SI group (Figure 3A, B). When compared with the CONT group, *S*. Infantis infection markedly decreased the level of butyrate in the colon (*P *= 0.020, Figure 3C). This decrease in butyrate level could be significantly reversed by pretreatment with CBB-MIX (*P *= 0.0007, Figure 3C). There was no difference in the level of valerate among all groups (Figure 3D). Isobutyrate and isovalerate concentrations were lower in the CBB-MIX group than those of the CONT group (*P *= 0.0003 and *P *= 0.006, Figure 3E, F). Moreover, compared with the CONT group, *S*. Infantis infection resulted in a marked decrease in the level of total SCFAs in the colon (*P *= 0.049), while this decrease was reversed by oral administration of CBB-MIX (*P* = 0.0005, Figure 3G).

### Butyrate attenuated cell damage by limiting the replication of cytosolic *S*. Infantis in Caco-2 cells

**Figure 4:**
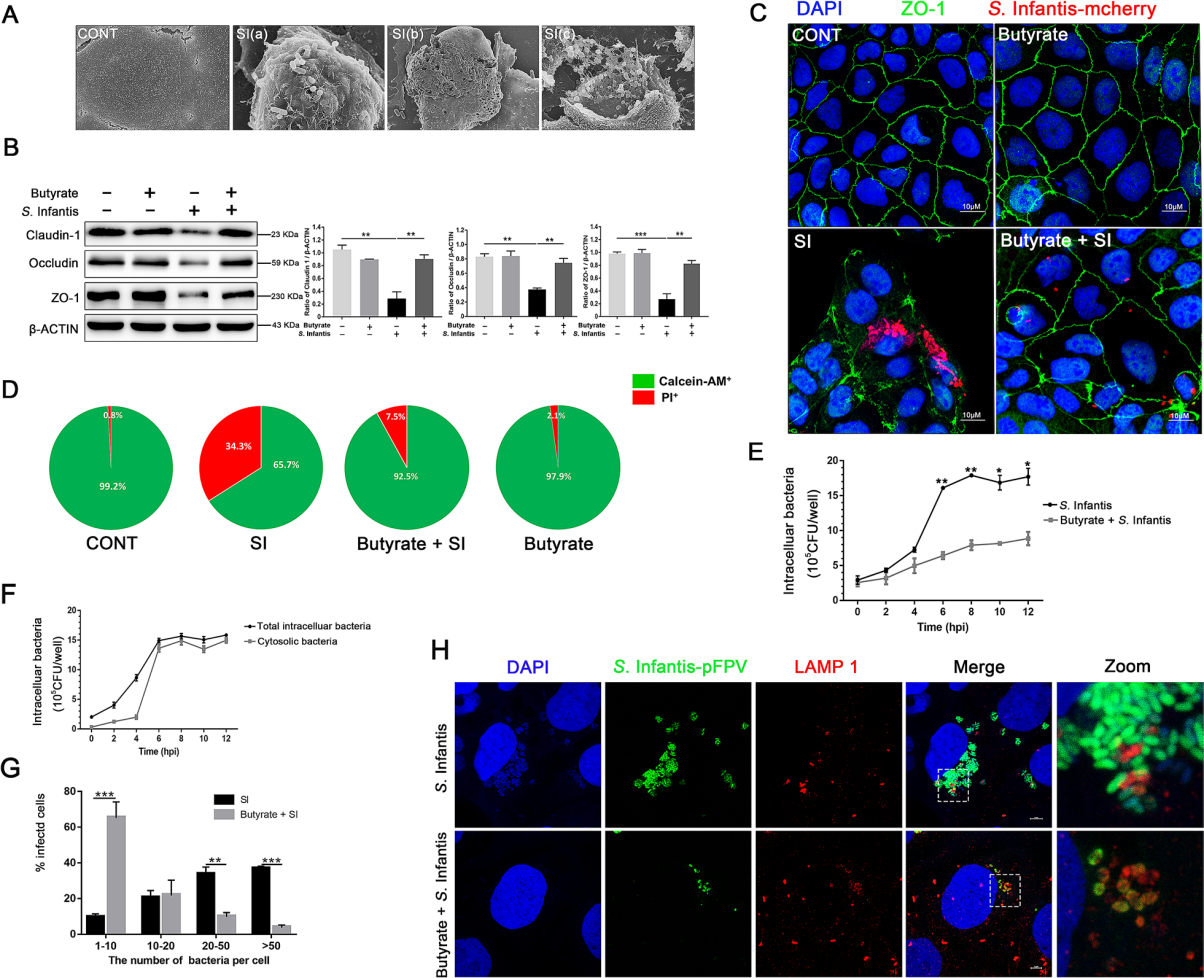
**Butyrate reduced cell damage by limiting replication of cytosolic*S*. Infantis Caco-2 cells. A** Observation of the apical surface of Caco-2 cell monolayer ultrastructure with scanning electron microscopy. SI(a), SI(b) and SI(c) showed that *S*. Infantis infected cell was bacteria-laden and these bacteria were extruded out of the monolayer. **B** Western blot analysis for the expression levels of claudin-1, occludin, and ZO-1 proteins. The right panel shows the quantitation analysis by Image J software (n = 3, **P < 0.01, ***P < 0.001). **C** Detection of the distribution and expression of ZO-1 (Green) in Caco-2 cells by immunofluorescence staining. Red: *Salmonella*. Blue: DAPI. Scale bar, 10 µm. **D** Calcein-AM/PI cell mortality test for *S*. Infantis-induced cell death rate. **E** Effect of butyrate on intracellular bacteria as assessed by the gentamicin protection assay. **F** The intracellular bacteria were assessed by the gentamicin protection assay, without (total intracellular bacteria, black curve) or with (cytosolic bacteria, gray curve) chloroquine. **G** The number of the infected cells containing 1 to 10, 10 to 20, 20 to 50, or more than 50 bacteria per cell at 8 hpi was measured using a fluorescence microscope. At least 100 cells were counted for each group and the percentage of cells containing the different numbers of bacteria is shown (mean ± SEM, n = 3), **P < 0.01, ***P < 0.001. **H** Caco-2 cells were infected with *S*. Infantis-pFPV (Green). Cells at 8 hpi were stained with LAMP-1 (Red) to label *S*. Infantis in SCV, while the cytosolic *S*. Infantis were green. “Zoom” denotes a magnified image of the white dotted area. Blue: DAPI. Scale bar, 10 µm.

The effect of butyrate on *S*. Infantis infection was explored in Caco-2 cells. *Salmonella* can proliferate within intestinal epithelial cells, and eventually a massive number of *Salmonella* escape from the infected cells into the intestinal cavity [12]. SEM images showed that *S*. Infantis punched holes in the outer membranes of Caco-2 cells and extruded toward the apical side, causing massive leakage of the cellular contents (Figure 4A). Immunoblot assays indicated that *S*. Infantis decreased occludin, claudin-1, and ZO-1 protein levels at 8 hpi compared with non-infected cells, while butyrate treatment preserved the high expression of these three tight junction proteins during *S*. Infantis infection (Figure 4B). Immunofluorescence assays further confirmed that *S*. Infantis caused the reduction of ZO-1 fluorescence area, and induced a disordered arrangement and aggregation of ZO-1 between cells, while butyrate reversed these phenomena (Figure 4C). The calcein-AM/PI cell mortality assay showed that compared with control cells, *S*. Infantis infection increased cell mortality by 34.3%, whereas co-incubation with butyrate lowered by this by 7.5% (Figure 4D). These results indicated that butyrate attenuated *S*. Infantis-induced cell damage.

We deduced that butyrate might limit the replication of intracellular *S*. Infantis, since the number of intracellular bacteria in the Butyrate + SI group was obviously less than that of the SI group (Figure 4C). Then, we quantified intracellular bacteria to explore whether butyrate reduced the replication of *Salmonella*, and adopted a gentamicin protection assay to define the percentage of infected cells containing different numbers of bacteria per cell. The results showed that butyrate did not attenuate the invasiveness of *S*. Infantis during the first 2 hpi (Figure 4E). Nevertheless, the replication of intracellular *S*. Infantis was restricted by butyrate between 6 to 12 hpi (Figure 4E). There are two distinct subpopulations of intracellular *Salmonella* in epithelial cells: cytosolic or SCV. Cytosolic *Salmonella* replicates to much higher than do SCV bacteria, a phenomenon known as hyper-replication [37, 38]. This suggested that butyrate might have specifically restricted this hyper-replication of cytosolic *Salmonella*. The chloroquine (CHQ) resistance assay determined that the quantity of cytosolic *Salmonella* comprise the majority of the total amount of intracellular bacteria at 6 hpi (Figure 4F). In the SI group, approximately 37% of total infected cells contained hyper-replicating bacteria, as recognized by the high numbers (> 50 bacteria per cell) of intracellular bacteria (Figure 4G). However, we found that in the Butyrate + SI group only 3% of infected cells contained hyper-replicating bacteria and 86% of infected cells contained less than 20 bacteria per cell (Figure 4G). We then distinguished these two subpopulations by examining the colocalization of these bacteria with LAMP1, a marker protein for the SCV membrane. These data showed that butyrate treatment did not change the quantity of SCV *Salmonella*, but that the cytosolic *Salmonella* quantity decreased significantly (Figure 4H). Collectively, the results suggested that butyrate limited the replication of cytosolic *S*. Infantis in Caco-2 cells.

### Butyrate suppressed *S*. Infantis-induced autophagy activation and alleviated lysosomal damage caused by *S*. Infantis in Caco-2 cells

**Figure 5:**
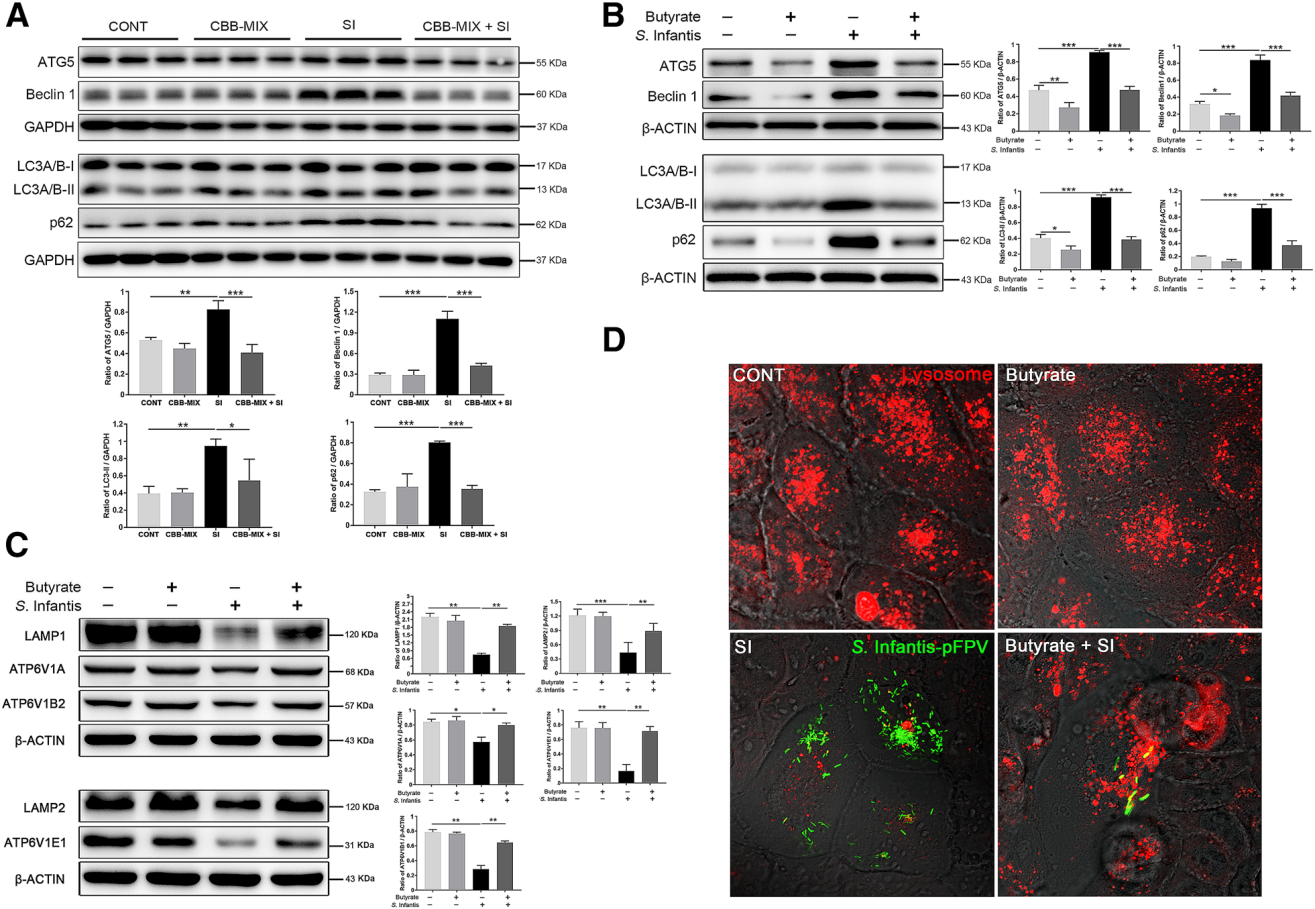
**Butyrate suppressed*S*. Infantis–induced activation of autophagy and alleviated lysosomal damage caused by*S*. Infantis in Caco-2 cells. A** Western blot analysis for the expression levels of ATG5, Beclin1, LC3-II, and p62 proteins in vivo. The lower panels show the quantitation analysis by Image J software. **B** Western blot analysis for the expression levels of ATG5, Beclin1, LC3-II, and p62 proteins in vitro. The right panel shows the quantitation analysis by Image J software. **C** Western blot analysis for the expression levels of LAMP1, LAMP2, ATP6V1A, ATP6V1B2, and ATP6V1E1 proteins in Caco-2 cells. **D** Immunofluorescence staining of the number of lysosomes in Caco-2 cells. Red: lysosomes. Green: *S*. Infantis. All data were presented as mean ± SEM (n = 6), *P < 0.05, **P < 0.01, ***P < 0.001.

Autophagy can serve as a natural defense mechanism to restrict or eliminate intracellular bacteria [21, 26, 30]. We next investigated the effects of *S*. Infantis on autophagy. Compared with the CONT group, *S*. Infantis infection increased the levels of ATG5, and Beclin1 proteins and the LC3-II/I ratio in the colon, but this increase was attenuated by treatment with the CBB-MIX (Figure 5A). Similarly, the increase in ATG5 and Beclin1 protein levels as well as the LC3-II/I ratio induced by *S*. Infantis at 8 hpi in Caco-2 cells was inhibited by butyrate (Figure 5B). In contrast, the protein level of p62 was upregulated both in vivo and in vitro (Figure 5A, B). An increased p62 protein level usually implies that the autophagy flux is blocked, especially at the stage of lysosome-mediated autophagic degradation [39]. We assumed that *S*. Infantis might disturb the function of lysosomes. Western blot assays showed that compared with control cells, *S*. Infantis infection downregulated the expression of the glycosylated membrane structural proteins LAMP1 and LAMP2, as well as ATP6V1A, ATP6V1B2, and ATP6V1E1. This decrease was alleviated by butyrate (Figure 5C). Furthermore, immunofluorescence images demonstrated that *S*. Infantis infection led to decreased numbers of lysosomes and these were smaller in size. Butyrate treatment prevented this situation (Figure 5D), suggesting that it suppressed *S*. Infantis-induced activation of autophagy and alleviated lysosomal damage caused by *S*. Infantis.

### Butyrate limited replication of cytosolic *S*. Infantis by inhibiting autophagy in Caco-2 cells

**Figure 6:**
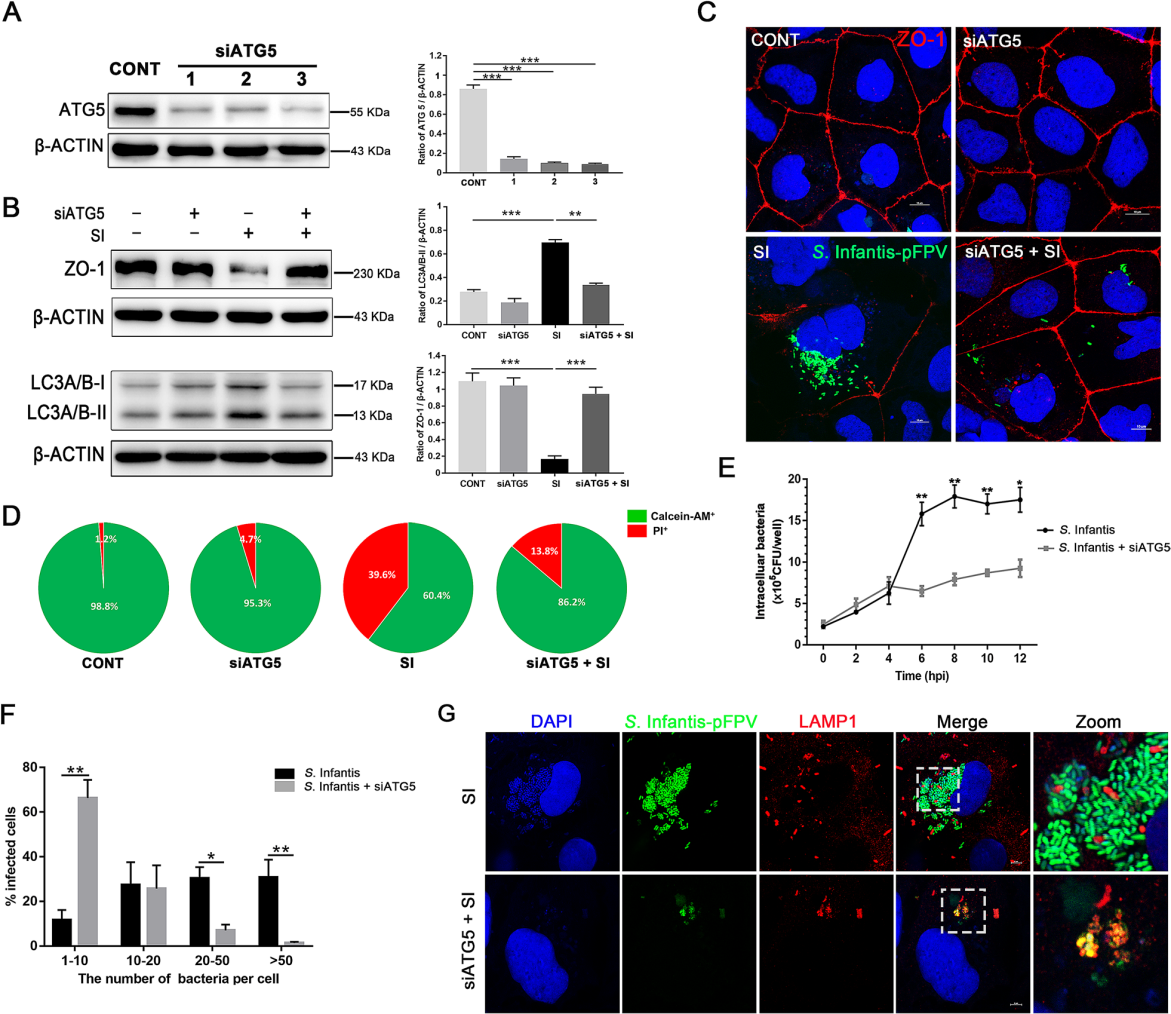
**Butyrate limited replication of cytosolic*S*. Infantis by inhibiting autophagy. A** Western blot analysis of Caco-2 cells treated with siRNA-targeting ATG5 (mean ± SEM, n = 3), ***P < 0.001. **B** Western blot analysis for the expression levels of ZO-1 and LC3 proteins. The right panel shows the quantitation analysis by ImageJ software. (mean ± SEM, n = 3), **P < 0.01, ***P < 0.001. **C** Detection of the distribution and expression of ZO-1 (Red) of Caco-2 cells by immunofluorescence staining. Blue: DAPI, Scale bar, 10 µm. **D** Cell death was measured using Calcein-AM/PI assay. **E** Effect of siATG5 on intracellular *S*. Infantis was assessed by the gentamicin protection assay. **F** The number of infected cells containing 1 to 10, 10 to 20, 20 to 50, or more than 50 bacteria per cell at 8 hpi was measured using a fluorescence microscope. At least 100 cells were counted for each group and the percentage of cells with the different numbers of bacteria is presented (mean ± SEM, n = 3), *P < 0.05, **P < 0.01. **G** Caco-2 cells were infected with *S*. Infantis-pFPV. Cells at 8 hpi were stained with LAMP-1 (Red) to label *S*. Infantis in SCV, while the cytosolic *S*. Infantis was green. “Zoom” denotes a magnified image of the white dotted area. Green: *S*. Infantis. Blue: DAPI. Scale bar, 10 µm.

We found that butyrate limited the replication of cytosolic *S*. Infantis and inhibited autophagy in Caco-2 cells during *S*. Infantis infection, but the role of autophagy during *S*. Infantis infection is still unclear. One recent study showed that autophagy facilitated *Salmonella* replication in HeLa cells [19]. We assumed that butyrate restricted *S*. Infantis replication through suppressing autophagy. To verify this hypothesis, we depleted ATG5 levels by siRNA. Western blot assays confirmed that siRNA effectively suppressed the expression of ATG5 (Figure 6A). Knockdown of ATG5 decreased *S*. Infantis-induced autophagy, which was confirmed by the decrease of the LC3-II protein level (Figure 6B). Compared with the untreated cells, *S*. Infantis infection reduced the level of ZO-1 protein at 8 hpi, but this reduction was reversed by knockdown of ATG5 (Figure 6B). Immunofluorescence analysis further confirmed that knockdown of ATG5 decreased the number of intracellular *S.* Infantis and ameliorated *S*. Infantis-induced disorder of ZO-1 protein arrangement and aggregation between cells (Figure 6C). Furthermore, ATG5 knockdown decreased the *S*. Infantis-induced cell mortality from 39.6 to 13.8% (Figure 6D).

We evaluated the effect of ATG5 knockdown on the replication of cytosolic *S*. Infantis. Similar to butyrate, ATG5 knockdown did not attenuate the invasiveness of *S*. Infantis, as assessed by the intracellular bacteria CFU during the first 2 hpi, but restricted the replication of intracellular *S*. Infantis between 6 to 12 hpi (Figure 6E). The proportion of cells with bacterial hyper-replication also decreased from 30.1 to 1.3% (Figure 6F). Indeed, the proportion of cells infected with less than 20 bacteria per cell in the siATG5 + SI group was higher than that of cells infected with *S*. Infantis alone (91.2% vs 39.5%). Additionally, the quantity of LAMP1^−^ bacteria (cytosolic) in the siATG5 + SI group markedly decreased compared with that in the SI group, while no visible differences were observed in the amount of LAMP1^+^ bacteria between the treatment groups (SCV) (Figure 6G). The results showed that butyrate limited the proliferation of cytosolic *S*. Infantis by inhibiting autophagy.

Taken together, these results suggest that treatment with CBB-MIX maintained the abundance of butyrate-producing bacteria in the colon and restored the butyrate level in weaned pigs infected with *S*. Infantis. Butyrate reduced cell damage by inhibiting autophagy to limit replication of cytosolic *S*. Infantis.

## Discussion

The CBB-MIX was composed of three strains: *L. johnsonii* CJ21, *B. subtilis* BS15, and *B. licheniformis* BL21, all of which were isolated from the intestinal contents of healthy pigs. *Lactobacillus* can improve the butyrate production capacity of butyrate producing bacteria in the intestinal tract [40]. Butyrate can activate the PPAR-γ signaling pathway of intestinal epithelial cells. This, in turn drives the energy metabolism of colon cells to transform into mitochondrial *β*-oxidation using butyric acid as the energy supply, consuming large amounts of oxygen. This form of energy metabolism is very important for the consumption of oxygen in the intestine. Only the continuous and stable mitochondrial *β*-oxidation in intestinal epithelial cells can ensure the formation of a physiological anoxic intestinal environment, thus ensuring a healthy microbiota structure dominated by specialized anaerobic bacteria [41, 42]. *Bacillus* can consume large amounts of oxygen in the intestine and accelerate the generation of an anaerobic environment in the gut [43–45]. It was reported that cyclic dipeptides containing Val-Leu and Val-Ile produced by *B. subtilis* C-3102 could serve as bifidogenic growth factors to increase the abundance of beneficial microorganisms related to the genus *Bifidobacterium* in a human colonic microbiota model culture system [46]. *B. subtilis* PB6 supplementation reduced *S.* Typhimurium level in the transverse colon of weaned Holstein steers at 48 h after *S*. Typhimurium infection [47]. In our previous work, we found that oral administration of a selected mixture of *B. licheniformis* and *B. subtilis* probiotics can regulate the microbial community structure in the colon and protect weaned pigs against Enterotoxigenic *E. coli* infection [48]. In addition, our previous studies using a piglet model with *S*. Infantis infection showed that a mixture of *Lactobacillus* and *Bacillus* reduced the number of *Salmonella* in the feces of piglets, while *L*. *johnsonii* administration reduced the degree of *Salmonella* colonization and expansion in various intestinal segments [30, 49]. Furthermore, it was found that *B.* subtilis B2A supplementation reduced the intestinal *Salmonella* burden in broiler chicks [50, 51].

In the present study, we found that the oral CBB-MIX treatment increased the abundance of butyrate-producing bacteria, including *Faecalibacterium*, *Ruminococcaceae*, *Lachnospiraceae*, and *Blautia* during the process of *S*. Infantis infection. These butyrate-producing bacteria can metabolize substrates to produce butyrate in the colon and most of them require an extremely strict anaerobic environment. Treatment with the CBB-MIX provided suitable conditions for the growth of these genera. Further, an elevated butyrate level was observed in the colon contents from the pigs pretreated with CBB-MIX. Butyrate is the terminal product of fermentation of specific anaerobic microbiota in the intestine which specifically down-regulates *Salmonella* pathogenicity island 1 gene expression [52]. Addition of sodium butyrate was reported to decrease *Salmonella* seroprevalence in fattening pigs [53], while administration of butyrate induced antimicrobial activity in intestinal macrophages in vivo and increased resistance to enteropathogens [54]. Our data suggested that treatment with the CBB-MIX enriched butyrate-producing bacteria and increased butyrate levels in the colon contents, thereby providing defense against *S*. Infantis infection in pigs.

Notably, butyrate suppresses autophagy at physiological concentrations [29]. In the present study, we found that butyrate limited the proliferation of cytosolic *S*. Infantis by suppressing autophagy, which alleviated cell damage. This conclusion challenged the current concept that autophagy protects the host against *Salmonella* infection [55–57]. A recent study suggested that HilA, a transcriptional regulator of SPI-1, can be acylated by butyrate, which weakens *Salmonella* Typhimurium invasion into epithelial cells [58]. In this study, we specifically excluded this factor by removing butyrate from the culture medium during the first 30 min of *S*. Infantis infection. The intracellular bacterial load assessment also showed that butyrate did not attenuate the invasiveness of *S.* Infantis. Subsequently, we showed that suppression of autophagy by ATG5 knockdown did inhibit the proliferation of cytosolic *S*. Infantis. We speculate that the time point of quantifying bacteria in cells may be an important consideration, as some studies have demonstrated that the autophagy receptor protein p62 limits *Salmonella* replication at 10 hpi [59]. Thus we quantified bacterial load at 8 hpi to avoid negative results due to cell separation or death at late time points. Consistent with our view that inhibition of autophagy limited *Salmonella* replication. Huang found the proliferation of *Salmonella* was slowed down when Rab1 was depleted, an essential GTPase for autophagy [18]. What is more convincing is that Yu et al. demonstrated that p62-dependent canonical autophagy was beneficial for the replication of cytosolic *Salmonella* in HeLa cells [19]. They observed a subpopulation of cytosolic *Salmonella* associated extensively with p62 or LC3 and the replication rate was much faster than that in SCV with a living cell station. It has been proved that cytosolic replication of *Salmonella* contributed to the majority of *Salmonella* net replication [8, 11, 38]. These intuitive evidence showed that autophagy favors the replication of *Salmonella*. Additionally, the fate of *Salmonella* is different after invading different types of cells. For example, there is no “free” cytosolic *Salmonella* within fibroblasts due to the effective autophagy response [60]. In macrophages, reactive oxygen and nitrogen species with strong activity can effectively kill invading bacteria [61, 62]. It is noteworthy that autophagy can also be “hijacked” by some other pathogens, like *Legionella*, *Coxiella*, and *Yersinia* [63]. Once fused with lysosomes to form autophagolysosomes, these organelles may serve as a source of nutrients for these intracellular pathogens. Autophagosomes may also act as a protective shelter for intracellular pathogens [64].

In conclusion, our data demonstrated that treatment with the CBB-MIX had strong properties in maintaining the composition and abundance of beneficial microbiota in the colon of piglets in the presence of *S*. Infantis, most especially the butyrate-producing bacteria. Butyrate is a metabolite of the beneficial microbiota, which can limit the proliferation of cytosolic *S*. Infantis in Caco-2 cells by inhibiting autophagy. The increasing situation of antibiotic resistance and emergence of new strains of *S*. Infantis indicate that a more thorough understanding of host–pathogen interactions and more novel antibiotic substitutes are urgently needed. Our research established a probiotic protection model based on a microbial metabolite, which provides a new perspective for better understanding of the protective activity of probiotics against pathogens.

## Data Availability

All data in this study are included in this article.

## Abbreviations

ASC: apoptosis-associated speck-like protein containing a CARD
Atg: autophagy-related gene
*B. subtilis*: *Bacillus subtilis*
*B. licheniformis*: *Bacillus licheniformis*
Caco: colon adenocarcinoma cells
CFU: colony-forming unit
CQ: chloroquine
DAPI: 4′,6′-Diamidino-2-phenylindole
hpi: hours post infection
LAMP: lysosomal-associated membrane protein
*L*. *johnsonii*: *Lactobacillus johnsonii*
LC3: microtubule-binding protein light chain 3
PBS: phosphate-buffered saline
*S*. Infantis: *Salmonella enterica* serovar Infantis
SCFAs: short chain fatty acids
SCV: *Salmonella*-containing vacuole
SEM: scanning electron microscopy
SQSTM1: sequestosome 1
ZO-1: zona occludens protein 1
